# Real-World Impact of Survival by Period of Diagnosis in Epithelial Ovarian Cancer Between 1990 and 2014

**DOI:** 10.3389/fonc.2019.00639

**Published:** 2019-08-06

**Authors:** San-Gang Wu, Jun Wang, Jia-Yuan Sun, Zhen-Yu He, Wen-Wen Zhang, Juan Zhou

**Affiliations:** ^1^Department of Radiation Oncology, Cancer Hospital, The First Affiliated Hospital of Xiamen University, Teaching Hospital of Fujian Medical University, Xiamen, China; ^2^State Key Laboratory of Oncology in South China, Department of Radiation Oncology, Sun Yat-sen University Cancer Center, Collaborative Innovation Center of Cancer Medicine, Guangzhou, China; ^3^Department of Obstetrics and Gynecology, The First Affiliated Hospital of Xiamen University, Teaching Hospital of Fujian Medical University, Xiamen, China

**Keywords:** ovarian neoplasms, general surgery, prognosis, SEER, time

## Abstract

**Introduction:** Although advances in surgical and chemotherapeutic approaches have improved management of epithelial ovarian cancer (EOC) in recent decades. The mortality of EOC over time remains controversial. The aim of this study was to assess the survival trends of EOC according to period of diagnosis using real-world data.

**Methods:** Patients with EOC diagnosed from 1990 to 2014 were included from the Surveillance, Epidemiology, and End Results database. The Kaplan-Meier method and multivariate Cox regression models were used to evaluate the trends in survival over time.

**Results:** We identified 59,763 patients diagnosed with EOC as follows: 6,586 (11.0%) in 1990–1994, 7,408 (12.4%) in 1995–1999, 15,348 (25.7%) in 2000–2004, 14,908 (24.9%) in 2005–2009, and 15,513 (26.0%) in 2010–2014. In the distant stage, the use of surgery decreased from 92.0% in 1990–1994 to 88.9% in 2010–2014. The use of chemotherapy increased from 67.4% in 1990–1994 to 75.0% in 2010–2014. The 5-year cause-specific survival (CSS) increased from 48.6% in 1990–1994 to 57.4% in 2010–2014 (*P* < 0.001). The 5-year overall survival (OS) increased from 42.7% in 1990–1994 to 51.7% in 2010–2014 (*P* < 0.001). The 5-year CSS and OS showed slight improvement in the localized stage (CSS, 91.9 vs. 93.1%; OS, 85.6 vs. 88.5%), and largely improved in the distant stage (CSS, 31.4 vs. 42.7%; OS, 26.7 vs. 37.4%) between 1990–1994 and 2010–2014. The multivariate analysis indicated that being diagnosed in the later years was related to better CSS and OS of EOC.

**Conclusion:** The trends in survival of EOC have improved over time, but net survival remains poor overall in distant-stage EOC.

## Background

Approximately 238,719 new patients with ovarian cancer are diagnosed each year worldwide. Although the survival trends between 1995–1999 and 2010–2014 were relatively constant in most countries, 5-year survival was still <50% for women diagnosed in 2010–2014 (1). Approximately 22,240 newly diagnosed ovarian cancer and 14,070 ovarian cancer-related deaths are expected in the United States (US) in 2018 (2). Epithelial ovarian cancer (EOC) is the most common histological subtype of ovarian cancer, and 90% of patients are diagnosed with EOC. However, 80% of patients have been diagnosed with advanced stage disease (2) due to lack of specificity and obvious symptoms (3, 4). In addition, there are still lack of sensitive and specific screening techniques and biomarkers for ovarian cancer (3–6).

A previous Surveillance, Epidemiology and End Results (SEER) study including EOC patients between 1973 and 1997, the 5-year relative survival was gradually increased in patients with localized (84% in 1973–1979 to 92% in 1990–1997), regional (49% in 1973–1979 to 77% in 1990–1997) and distant (17% in 1973–1979 to 27% in 1990–1997) stage (7). The significantly increased the survival in regional stage may be due to the use of paclitaxel-containing chemotherapy in clinical practice. In a recent ovarian cancer statistics between 2007 to 2013 from US, the 5-year survival rate was 89 and 71% in stage I and II EOC patients, respectively, while it has significantly decreased to 41 and 20% in those with stage III and IV disease, respectively (2). Although the above three studies have different definitions of staging system, the ovarian cancer mortality was slowly decreasing in recent decades, which may be related to its unknown etiology, lack of effective diagnostic methods in the early disease process, and lack of effective treatment.

Advances in surgical and chemotherapeutic approaches have improved management of EOC in recent decades. The cytoreductive surgery (debulking) and paclitaxel-containing chemotherapy were introduced into the clinical practice of EOC in the late 1960s and early 1990s, respectively. In the current clinical practice, the standard treatment for early-stage EOC is complete surgical staging procedure including examination of the abdominal cavity, omentectomy, several prescribed biopsies, and thorough pelvic and para-aortic lymph node sampling. Debulking surgery in combination with neoadjuvant or adjuvant platinum/taxane-based chemotherapy has become the standard treatment for patients with advanced EOC (8, 9). The main goal of all these therapeutic changes is to improve the prognosis. However, the results of several population-based studies on survival trends over time in EOC are inconsistent (10–15), which may be due to the different period of diagnosis, heterogeneity of the population, and different treatment strategies. A SEER study included EOC patients between 1973 and 1997, reported a gradual increase in survival over time. However, the 5-year overall survival (OS) only increased slightly from 40% in 1980–1989 to 45% in 1990–1997 (7). In addition, another SEER study included Hispanic EOC patients during 1992–2013, and the results showed no significantly difference in survival outcomes among three periods of diagnosis 1992–1999, 2000–2006, and 2007–2013 (16). There are lacking studies regarding to the changes in the survival rates of EOC in the recent three decades over time. Due to improvements in diagnostic techniques, surgery, treatment, and individualized care, it is hypothesized that the survival rate of EOC may be further improved in recent decades. The purpose of the present study was to assess the survival trends of EOC between 1990 and 2014 using real-world data from SEER program.

## Materials and Methods

### Patients

Data on patients diagnosed with EOC from 1990 to 2014 were analyzed using the SEER 18 Regs Research Data of the National Cancer Institute (17). This cancer-registration database contains data on cancer incidence, demographic and clinicopathologic characteristics, first course of treatment, and survival of approximately 28% of the US population. Patients without a positive histology or with a prior malignancy diagnosis were excluded. The use of data from the SEER database was exempt from the approval process of the Institutional Review Board because its patient-related information is de-identified.

### Measures

Demographic, clinicopathologic, treatment variables, and vital status were included as follows: age, year of diagnosis, race/ethnicity, tumor grade, SEER stage, histological subtypes, surgery, chemotherapy, and marital status at diagnosis. Survival trends were examined over five time periods: 1990–1994, 1996–1999, 2000–2004, 2005–2009, and 2010–2014. Tumor grade was classified as well-differentiated, moderately differentiated, poorly differentiated, undifferentiated, or unknown tumor grade. The histologic subtypes were classified as serous, mucinous, endometrioid, and clear-cell. The SEER staging system corresponds to the commonly used International Federation of Gynecology and Obstetrics (FIGO) staging system, as follows: localized (I-A, I-B, I-not otherwise specified [NOS]), regional (FIGO I-C, II-A, II-B, II-C, II-NOS), distant (FIGO III-A, III-B, III-C, III-NOS, IV) (18).

### Statistical Analysis

The chi-square test and one-way analysis of variance were used to compare patients' demographic, clinicopathologic, and treatment variables over the five periods. Kaplan–Meier analyses for 5-year cause-specific survival (CSS) and OS were performed using the log-rank test. CSS was defined as the time from the date of the initial diagnosis to the date of the ovarian cancer-related death. OS was defined as the time from the date of diagnosis to the date of death or last follow-up. The Cox proportional hazard was used for multivariate analyses. We included the following variables in the multivariate Cox proportional hazard models: age, race/ethnicity, grade, SEER stage, histological subtypes, surgery, chemotherapy, marital status, and years of diagnosis. Trends in survival were analyzed separately for localized, regional, and distant stages. All analyses were performed using SPSS version 22.0 (IBM Corporation, Armonk, USA), and *P* < 0.05 was considered significant.

## Results

### Patient Characteristics

We identified 59,763 EOC patients in this study, including 6,586 (11.0%), 7,408 (12.4%), 15,348 (25.7%), 14,908 (24.9%), and 15,513 (26.0%) patients diagnosed in 1990–1994, 1995–1999, 2000–2004, 2005–2009, and 2010–2014, respectively (Table 1). Patients with poorly differentiated/undifferentiated disease increased from 43.1% in 1990–1994 to 56.1% in 2010–2014 (*P* < 0.001). Patients with regional stage disease increased from 6.2% in 1990–1994 to 11.1% in 2010–2014 (*P* < 0.001). Serous EOC was the most common histological subtype; the number of serous and clear-cell subtypes increased during the study period, whereas the number of endometrioid and mucinous subtypes decreased during the study period (*P* < 0.001).

**Table 1:** Baseline characteristics of patients between 1990 and 2014 by period of diagnosis.

**Variables**	***N***	**1990–1994 (%)**	**1995–1999 (%)**	**2000–2004 (%)**	**2005–2009 (%)**	**2010–2014 (%)**	***P***
**Age (years)**							
Mean (years) (SD)	60 (13.8)	60.4 (14.6)	60.1 (14.1)	60.0 (13.9)	60.2 (13.7)	60.2 (13.4)	0.739
<20	128	12 (0.2)	12 (0.2)	23 (0.1)	37 (0.2)	44 (0.3)	<0.001
20–39	3,845	612 (9.3)	527 (7.1)	995 (6.5)	853 (5.7)	858 (5.5)	
40–59	24,831	2,330 (35.4)	3,018 (40.7)	6,563 (42.8)	6,424 (43.1)	6,496 (41.9)	
60–79	26,047	3,101 (47.1)	3,253 (43.9)	6,492 (42.3)	6,291 (42.2)	6,910 (44.5)	
>79	4,912	531 (8.1)	598 (8.1)	1,275 (8.3)	1,303 (8.7)	1,205 (7.8)	
**Race**							
Non-hispanic white	45,028	5,386 (81.8)	5,737 (77.4)	11,947 (77.8)	11,074 (74.3)	10,884 (70.2)	<0.001
Non-hispanic black	3,701	352 (5.3)	416 (5.6)	916 (6.0)	929 (6.2)	1,088 (7.0)	
Hispanic	5,943	398 (6.0)	588 (7.9)	1,371 (8.9)	1,620 (10.9)	1,966 (12.7)	
Other	5,091	450 (6.8)	667 (9.0)	1,114 (7.3)	1,285 (8.6)	1,575 (10.2)	
**Grade**							
Well-differentiated	5,536	675 (10.2)	810 (10.9)	1,401 (9.1)	1,281 (8.6)	1,369 (8.8)	<0.001
Moderately differentiated	10,728	1,445 (21.9)	1,698 (22.9)	3,066 (20.0)	2,515 (16.9)	2,004 (12.9)	
Poorly/undifferentiated	30,515	2,836 (43.1)	3,528 (47.6)	7,552 (49.2)	7,895 (53.0)	8,704 (56.1)	
Unknown	12,984	1,630 (24.7)	1,372 (18.5)	3,329 (21.7)	3,217 (21.6)	3,436 (22.1)	
**SEER stage**							
Localized	13,165	1,531 (23.2)	1,691 (22.8)	3,194 (20.8)	3,273 (22.0)	3,476 (22.4)	<0.001
Regional	5,289	408 (6.2)	517 (7.0)	1,255 (8.2)	1,394 (9.4)	1,715 (11.1)	
Distant	420,425	4,533 (68.8)	5,054 (68.2)	10,568 (68.9)	10,101 (67.8)	10,169 (65.6)	
Unknown	884	114 (1.7)	146 (2.0)	331 (2.2)	140 (0.9)	153 (1.0)	
**Histological subtypes**							
Serous	40,298	4,196 (63.7)	4,727 (63.8)	10,270 (66.9)	10,299 (69.1)	10,806 (69.7)	<0.001
Endometrioid	9,054	1,059 (16.1)	1,280 (17.3)	2,470 (16.1)	2,098 (14.1)	2,147 (13.8)	
Mucinous	5,815	927 (14.1)	881 (11.9)	1,490 (9.7)	1,287 (8.6)	1,230 (7.9)	
Clear cell	4,596	404 (6.1)	520 (7.0)	1,118 (7.3)	1,224 (8.2)	1,330 (8.6)	
**Surgery**							
No	3,993	420 (6.4)	423 (5.7)	939 (6.1)	985 (6.6)	1,226 (7.9)	<0.001
Yes	55,605	6,155 (93.5)	6,978 (94.2)	14,363 (93.6)	13,875 (93.1)	14,234 (91.8)	
Unknown	165	11 (0.1)	7 (0.1)	46 (0.3)	48 (0.3)	53 (0.3)	
**Chemotherapy**							
No/unknown	17,926	2,150 (32.6)	2,279 (30.8)	5,139 (33.5)	4,474 (30.0)	3,884 (25.0)	<0.001
Yes	41,837	4,436 (67.4)	5,129 (69.2)	10,209 (66.5)	10,434 (70.0)	11,629 (75.0)	
**Marital status**							
Unmarried	25,697	2,927 (44.4)	3,168 (42.8)	6,456 (42.1)	6,451 (43.3)	6,695 (43.2)	<0.001
Married	31,998	3,498 (53.1)	4,078 (55.0)	8,367 (54.5)	7,964 (53.4)	8,091 (52.2)	
Unknown	2,068	161 (2.4)	162 (2.2)	525 (3.4)	493 (3.3)	727 (4.7)	

*SD, standard deviation; SEER, surveillance, epidemiology and end results*.

### Trends in Treatment

The proportion of patients who received surgical treatment decreased from 93.5% in 1990–1994 to 91.8% in 2010–2014 (*P* < 0.001). No significant difference was found over time in the proportion of patients in the localized (*P* = 0.140) and regional stages (*P* = 0.099) who received surgical treatment. However, the use of surgery decreased from 92.0% in 1990–1994 to 88.9% in 2010–2014 in the distant stage (*P* < 0.001). The use of chemotherapy increased from 67.4% in 1990–1994 to 75.0% in 2010–2014. The use of chemotherapy in the localized stage increased from 34.0% in 1990–1994 to 49.7% in 2010–2014 (*P* < 0.001). The use of chemotherapy in the regional stage increased from 70.6% in 1990–1994 to 78.5% in 2010–2014 (*P* < 0.001), and it increased in the distant stage from 78.7% in 1990–1994 to 83.5% in 2010–2014 (*P* < 0.001).

### Survival Analysis

The median follow-up was 40 months (range = 0–311 months). A total of 36,031 patients died, including 28,852 patients from ovarian cancer. The 5-year CSS and OS were 53.7 and 48.0%, respectively, and the median CSS and OS were 72.0 and 56.0 months, respectively.

**Figure 1:**
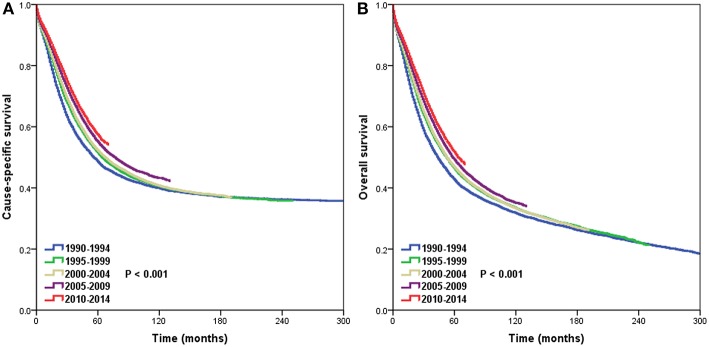
Trends in cause-specific survival **(A)** and overall survival **(B)** over time.

**Figure 2:**
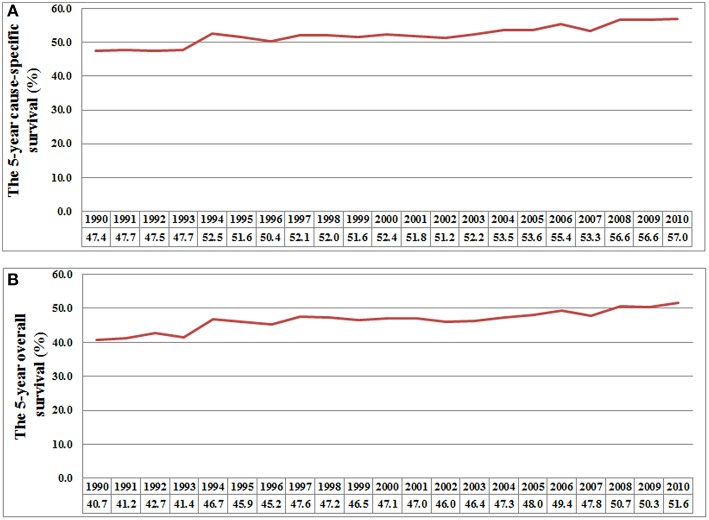
The 5-year cause-specific survival **(A)** and overall survival **(B)** by each year of diagnosis from 1990 to 2010.

The 5-year CSS rates were 48.6, 51.6, 52.2, 55.1, and 57.4% for patients diagnosed between 1990–1994, 1995–1999, 2000–2004, 2005–2009, and 2010–2014, respectively (*P* < 0.001) (Figure 1A), and the 5-year OS rates were 42.7, 46.4, 46.8, 49.3, and 51.7%, respectively (*P* < 0.001) (Figure 1B). Figure 2 lists the 5-year CSS (Figure 2A) and OS (Figure 2B) by year of diagnosis from 1990 to 2010. There was a 9.6% and a 10.9% absolute increase in the CSS and OS from 1990 to 2010, respectively.

### Multivariate Analysis of Prognostic Factors

The results multivariate analysis showed that diagnoses in later years were associated with better CSS and OS (Table 2). Age, race/ethnicity, grade, SEER stage, histological subtypes, surgery, chemotherapy, and marital status were also the independent prognostic factors of CSS and OS.

**Table 2:** Multivariate prognostic analyses of the entire cohort.

**Variables**	**CSS**			**OS**		
	**HR**	**95% CI**	***P***	**HR**	**95% CI**	***P***
**Age (years)**						
<20	1			1		
20–39	0.993	0.677–1.456	0.970	1.156	0.805–1.662	0.432
40–59	1.418	0.971–2.070	0.071	1.895	1.323–2.713	<0.001
60–79	1.893	1.296–2.763	0.001	2.865	2.001–4.102	<0.001
>79	3.076	2.104–4.497	<0.001	5.060	3.530–7.251	<0.001
**Race**						
Non-hispanic white	1			1		
Non-hispanic black	1.191	1.136–1.248	<0.001	1.256	1.205–1.309	<0.001
Hispanic	0.940	0.901–0.981	0.004	0.990	0.953–1.027	0.584
Other	0.903	0.862–0.947	<0.001	0.927	0.889–0.967	<0.001
**Grade**						
Well-differentiated	1			1		
Moderately differentiated	1.960	1.824–2.106	<0.001	1.530	1.449–1.616	<0.001
Poorly/undifferentiated	2.350	2.194–2.518	<0.001	1.814	1.722–1.911	<0.001
Unknown	2.126	1.979–2.284	<0.001	1.682	1.593–1.776	<0.001
**SEER stage**						
Localized	1			1		
Regional	3.184	2.958–3.428	<0.001	2.069	1.957–2.187	<0.001
Distant	8.436	7.954–8.947	<0.001	4.790	4.597–4.992	<0.001
Unknown	4.272	3.826–4.772	<0.001	2.417	2.205–2.648	<0.001
**Histological subtypes**						
Serous	1			1		
Endometrioid	0.656	0.627–0.686	<0.001	0.744	0.717–0.772	<0.001
Mucinous	1.130	1.072–1.192	<0.001	1.240	1.188–1.295	<0.001
Clear cell	1.093	1.036–1.153	0.001	1.012	0.965–1.062	0.617
**Surgery**						
No	1			1		
Yes	0.385	0.368–0.402	<0.001	0.372	0.358–0.386	<0.001
Unknown	0.546	0.429–0.694	<0.001	0.612	0.501–0.749	<0.001
**Chemotherapy**						
No/unknown	1			1		
Yes	0.873	0.848–0.898	<0.001	0.800	0.780–0.820	<0.001
**Marital status**						
Unmarried	1			1		
Married	0.873	0.852–0.894	<0.001	0.845	0.827–0.864	<0.001
Unknown	0.815	0.759–0.875	<0.001	0.835	0.785–0.888	<0.001
**Year of diagnosis**						
1990–1994	1			1		
1995–1999	0.921	0.882–0.962	<0.001	0.923	0.889–0.959	<0.001
2000–2004	0.864	0.832–0.898	<0.001	0.877	0.848–0.907	<0.001
2005–2009	0.772	0.742–0.803	<0.001	0.801	0.773–0.830	<0.001
2010–2014	0.671	0.642–0.702	<0.001	0.699	0.672–0.727	<0.001

*CSS, cause-specific survival; CI, confidence interval; HR, hazard ratio; OS, overall survival; SEER, surveillance, epidemiology, and end results*.

When adjusted by age, race, grade, histological subtypes, surgery, chemotherapy, and marital status, patients in the localized stage diagnosed in 2005**–**2009 (CSS hazard ratio [HR], 0.828; 95% confidence interval [CI], 0.694–0.988, *P* = 0.036; OS HR, 0.876; 95% CI 0.779–0.986, *P* = 0.028) and 2010–2014 (CSS HR, 0.668; 95% CI 0.538–0.831, *P* < 0.001; OS HR, 0.783; 95% CI 0.670–0.914, *P* = 0.002) had a better CSS and OS than those diagnosed in 1990–1994. The CSS increased over time among patients in the regional stage, with patients diagnosed in 2005–2009 (HR, 0.745; 95% CI, 0.641–0.866, *P* < 0.001) and 2010–2014 (HR, 0.802; 95% CI, 0.680–0.947, *P* = 0.009) having better OS compared to those diagnosed in 1990–1994. Patients diagnosed in later years had a higher CSS and OS in the distant stage when compared to patients diagnosed in earlier years (Table 3).

**Table 3:** The 5-year cause-specific survival, overall survival, and adjusted hazard ratios for epithelial ovarian cancer patients by period of diagnosis according to SEER stage.

**Variables**	**CSS**					**OS**				
	**5-year (%)**	***P***	**HR**	**95% CI**	***P***	**5-year (%)**	***P***	**HR**	**95% CI**	***P***
**Localized**										
1990–1994	91.9	0.265^*^	1			85.6	0.064^*^	1		
1995–1999	91.9	0.210^#^	0.982	0.878–1.180	0.845	87.2	0.309^#^	0.978	0.877–1.090	0.685
2000–2004	91.9	0.141^†^	0.910	0.771–1.075	0.269	87	0.234^†^	0.911	0.82101.012	0.081
2005–2009	91.9	0.096^‡^	0.828	0.694–0.988	0.036	86.8	0.121^‡^	0.876	0.779–0.986	0.028
2010–2014	93.1		0.668	0.538–0.831	<0.001	88.5		0.783	0.670–0.914	0.002
**Regional**										
1990–1994	66.4	0.022^*^	1			59.7	0.089^*^	1		
1995–1999	72.5	0.298^#^	0.806	0.659–0.985	0.035	65.0	0.430^#^	0.870	0.739–1.023	0.093
2000–2004	73.8	0.246^†^	0.794	0.669–0.943	0.009	67.1	0.412^†^	0.874	0.756–1.009	0.067
2005–2009	75.2	0.104^‡^	0.670	0.562–0.800	<0.001	69.2	0.195^‡^	0.745	0.641–0.866	<0.001
2010–2014	72.9		0.756	0.625–0.916	0.004	66.9		0.802	0.680–0.947	0.009
**Distant**										
1990–1994	31.4	<0.001^*^	1			26.7	<0.001^*^	1		
1995–1999	35.4	<0.001^#^	0.915	0.873–0.958	<0.001	31.1	<0.001^#^	0.906	0.868–0.946	<0.001
2000–2004	36.9	<0.001^†^	0.858	0.824–0.894	<0.001	32.1	<0.001^†^	0.865	0.833–0.898	<0.001
2005–2009	39.7	<0.001^‡^	0.773	0.742–0.807	<0.001	34.4	<0.001^‡^	0.791	0.761–0.822	<0.001
2010–2014	42.7		0.661	0.630–0.692	<0.001	37.4		0.679	0.650–0.709	<0.001

*CSS, cause-specific survival; CI, confidence interval; HR, hazard ratio; OS, overall survival*.

* *Indicates Kaplan–Meier analyses of 5-year survival and comparisons among the five time periods using the log-rank test*.

# *Indicates Kaplan–Meier analyses of 5-year survival and comparisons using the log-rank test among the last four periods (1995–1999, 2000–2004, 2005–2009, and 2010–2014)*.

† *Indicates the Kaplan–Meier analyses of 5-year survival and comparisons using log-rank test among three periods (2000–2004, 2005–2009, and 2010–2014)*.

‡ *Indicates the Kaplan–Meier analyses of 5-year survival and comparisons using the log-rank test on the last two periods (2005–2009 and 2010–2014)*.

**Figure 3:**
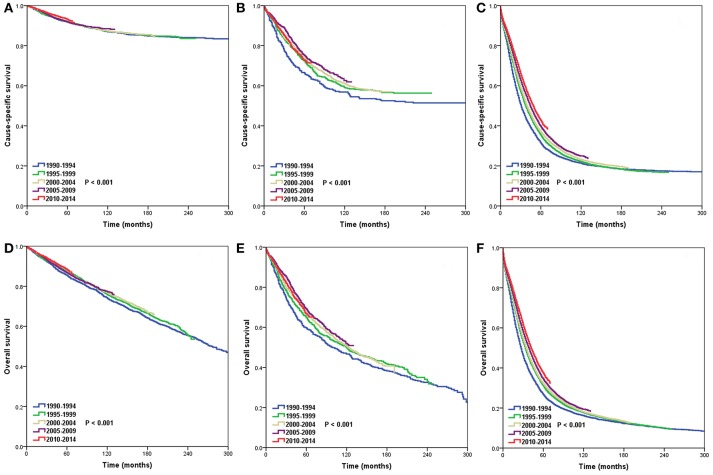
Trends in cause-specific survival (CSS) and overall survival (OS) in the localized (CSS, **A**; OS, **D**), regional (CSS, **B**; OS, **E**), and distant stages (CSS, **C**; OS, **F**) over time.

The 5-year CSS and OS by period of diagnosis according to SEER stage are presented in Table 3. The period of diagnosis had no effect on the CSS and OS of patients in the localized stage (Figures 3A,D). The CSS significantly improved in the regional state after 1995–2014 compared to 1990–1994, while it did not differ significantly from 1995 to 2014. The OS in the regional stage during the study period was not significantly different (Figures 3B,E). However, the CSS and OS gradually increased from 1990 to 2014 in the distant stage (Figures 3C,F).

## Discussion

The purpose of the present study was to assess trends in survival outcomes among EOC patients between 1990 and 2014. We found a 9.6% improvement in the 5-year CSS and a 10.9% improvement in the 5-year OS between 1990 and 2010, and survival showed the greatest increase among patients in the distant stage. The incidence of EOC has gradually decreased in recent decades (2). However, the absolute number of EOC cases in our study increased and was relatively stable after 2000, which may be related to changes in the histological diagnostic patterns of EOC (19). The increase in the number of cases classified as EOC may be due to better coding and recording of ovarian cancer morphology by cancer registries (20). We found approximately 25% of patients were diagnosed in the localized stage, and this proportion did not change over time, which may be related to the lack of reliable and effective screening methods to diagnosis early-stage EOC (3–6). Therefore, although the incidence of EOC is declining, there is a need to explore effective screening methods, including mathematical modeling, cancer-specific biomarkers, and real-time imaging to detect it earlier (21).

There were also several SEER studies attempted to answer similar questions, but the results were inconsistent (7, 12, 14, 16). A SEER study included patients from 1973 to 1997, and the results showed improved outcomes in localized, regional, and distant stage EOC over time (7). Two SEER studies included patients in later years (1988–2011 and 1992–2013) showed comparable outcomes during the periods of diagnosis (12, 16). However, a study only included patients with stage I-II disease (12), and another study only included Hispanic women (16), which could not represent the general population. A recent SEER study included patients from 2002 to 2011, and the results showed improved outcomes in patients with stage IV EOC, but not in patients with stage I–III disease (14). However, the information regarding sample of patients, surgery and chemotherapy were not included in this study. Although studies from Canadian and France population showed an improved outcome over time. A large cohort from Netherlands Cancer Registry indicated that long-term outcome has not improved of EOC in the last 25 years (13). As listed in Table 4, there were significantly differences in the inclusion criteria and patterns of treatment in the above-mentioned studies, which may be contributed to the inconsistent results. In addition, the diagnostic techniques, postoperative care, and palliative care may also have different effects on the survival outcome for patients.

**Table 4:** The similarities and differences in present study and previous studies.

**References**	**Period of diagnosis**	**Population**	**No. of patients**	**Stage and histology**	**Surgery**	**Chemotherapy**	**Survival trends by period of diagnosis**
Barnholtz-Sloan et al. (7)	1973–1997	SEER database	32,845	Localized, regional, distant, and unknown stage EOC	79.0%	NA	Localized stage: 5-year OS 84, 88, and 92% in patients diagnosed in 1973–1979, 1980–1989, and 1990–1997, respectively. Regional stage: 5-year OS 49, 65, and 77% in patients diagnosed in 1973–1979, 1980–1989, and 1990–1997, respectively. Distant stage: 5-year OS 17, 22, and 27% in patients diagnosed in 1973–1979, 1980–1989, and 1990–1997, respectively.
Chan et al. (12)	1988–2001	SEER database	8,372	Stage I–II EOC	94.9%	NA	Comparable disease-specific survival by 1988–1992, 1993–1997, and 1998–2001 (*P* = 0.076).
Tan et al. (15)	1979–2001	New York State	NA	NA	NA	NA	Ovarian cancer death rates relatively constant over time.
Akhtar-Danesh et al. (10)	1992–2005	Canadian population	7,771	EOC	NA	NA	2- and 5-year relative survival improved over the period of 1992–2005.
Trétarre et al. (11)	1989–2010	France	12,645	NA	NA	NA	5-year net survival 36, 35, 42, and 44% in patients diagnosed in 1989–1993, 1994–1998, 1999–2004, and 2005–2010, respectively.
Warren et al. (14)	2002–2011	SEER database	NA	Stage I–IV EOC	NA	NA	There was an increase in 2-year cause-specific survival in stage IV EOC from 2002 to 2011, but 2-year cause-specific survival remained stable in patients with stage I–III diseases.
Timmermans et al. (13)	1989–2014	Netherlands Cancer Registry	32,540	Stage I–IV EOC	98.2% (early stage) 71.7% (advanced stage)	38.8% (early stage) 79.3% (advanced stage)	5-year OS 31.1, 31.7, 35.1, 34.6, and 33.7% in patients diagnosed in 1989–1993, 1994–1998, 1999–2003, 2004–2008, and 2009–2014, respectively.
Chen et al. (16)	1992–2013	SEER Hispanic women	7,780	Localized, regional, distant, and unknown stage EOC	78.3%	NA	No significantly difference in all-cause and ovarian cancer-specific survival in patients diagnosed in 1992–1999, 2000–2006, and 2007–2013.
Present studyNA	1990–2014	SEER database	59,763	Localized, regional, distant, and unknown stage EOC	93.0%	70.0%	Survival of women with EOC has improved over time.

*EOC, epithelial ovarian cancer; NA, not available; SEER, Surveillance, Epidemiology, and end results; OS, overall survival*.

Surgery remains the standard treatment for localized stage EOC. Our multivariate analysis found survival outcomes were better in 2005–2014 compared to 1990–1994. However, a slight improvement was found in CSS and OS; the 5-year CSS was 91.9% in 1990–1994 and in 2005–2009, and only a 1.2% absolute increase in OS was found between 1990–1994 and 2005–2009. A SEER study of patients with stages I-II EOC between 1988 and 2001 found the year of diagnosis was associated with better disease-specific survival in patients who received surgery that excluded lymphadenectomy, but it was not associated with better disease-specific survival in patients who received surgery that included lymphadenectomy (12). Therefore, the use of staging procedures may impact the role of period of diagnosis in EOC outcomes.

In our study, the CSS of patients diagnosed after 1995 significantly improved compared to those diagnosed in 1990–1994. This improvement might be related to the introduction of taxanes to clinical practice in EOC (22). Moreover, the proportion of optimal debulking surgeries for residual tumors ≤1 cm has increased in the past two decades, which may also account for improved outcomes in the regional disease stage between 1990–1994 and 1998–2014; it did not differ significantly between 1995 and 2015 (23, 24).

Our findings showed that the CSS and OS of patients in the distant stage improved over time. The reasons for improved survival may involve advances in chemotherapy, the introduction of carboplatin and paclitaxel for first-line treatment, the use of backup regimens for non-responsive tumors, and progress in developing treatments, such as PAPR-1 inhibitor and PD-L1 inhibitor (8, 25–29). In addition, there is also evidence of more accurate surgical staging procedures, more extensive tumor debulking surgery, and more accurate patient selection for secondary cytoreduction. Moreover, it is also possible the dissemination of evidence-based practices has had an impact on improving survival outcomes (8). Finally, significant improvements in survival outcomes may reflect the impact of new government-administrative strategies, such as having oncology multidisciplinary teams provide optimal treatment and care. Although gynecologic-oncologist consultations have increased in ovarian cancer over time, <40% of patients who saw a gynecologic oncologist received guidelines pertaining to surgery and chemotherapy (14). A recent review of outcomes of the management of ovarian cancer found gynecologic oncologists in specialized hospitals consistently performed staging and debulking surgery better (30). Therefore, collaboration among patients, gynecologic oncologists, and institutions is crucial.

This study has many strengths. The primary strength of this study is that we used high quality, nationwide, population-based data to gain greater insight into the survival patterns of EOC patients over a long period of time. In addition, a large sample of EOC patients was included, thus providing sufficient power to detect the smallest differences in relative survival for EOC patients.

This study also has several limitations. First, the major limitation of this study was the bias of observational studies and the unavailability of several variables including performance status, comorbidities, smoking status, and obesity, which may be the possible confounders for the modeling of outcome for EOC. In addition, the completeness of chemotherapy and surgery were also not recorded in the SEER database, which might have influenced the results. Second, schedules, agents, and the number of courses of chemotherapy, target therapy, interval debulking surgery, and the extent of residual tumors after cytoreductive surgery (available data since 2010) were not recorded in the SEER database. Third, the SEER program does not include treatment data beyond 4 months after diagnosis, and the treatment strategy after disease recurrence are also not recorded. Moreover, a high rate of under-reporting chemotherapy has been found in the SEER database (31). Finally, the median follow-up in patients diagnosed after 2010 was significantly shorted compared to patients who diagnosed before 2010. However, approximately two-thirds of patients have been diagnosed as distant stage, which was associated with poor prognosis. Therefore, although the follow-up time of our study needs to be further extended to confirm the results, we believe that our study is still representative to reflect survival trend of EOC over time.

## Conclusion

In conclusion, our results indicate the survival of women with EOC has improved over time. This study indicates that advances in the management of patients with EOC have improved survival, but net survival remains generally poor in distant-stage EOC. There is a need to explore additional treatment strategies to improve the survival of patients with EOC.

## Data Availability

Publicly available datasets were analyzed in this study. This data can be found here: www.seer.cancer.gov (access number: 11025-Nov2016).

## Ethics Statement

This study was exempt from the approval processes of the Institutional Review Boards because the SEER database patient information is de-identified.

## Author Contributions

JZ, S-GW, and W-WZ are lead authors who participated in data collection, manuscript drafting, table/figure creation, and manuscript revision. JW, Z-YH, and J-YS are senior authors who aided in drafting the manuscript and manuscript revision. W-WZ and JZ are the corresponding authors who initially developed the concept and drafted and revised the manuscript. All authors read and approved the final manuscript.

### Conflict of Interest Statement

The authors declare that the research was conducted in the absence of any commercial or financial relationships that could be construed as a potential conflict of interest.

## References

[1] Allemani C Matsuda T Di Carlo V Harewood R Matz M Nikšić M. Global surveillance of trends in cancer survival 2000–14 (CONCORD-3): analysis of individual records for 37 513 025 patients diagnosed with one of 18 cancers from 322 population-based registries in 71 countries. Lancet. (2018) 391:1023–75. 10.1016/S0140-6736(17)33326-3 29395269 PMC5879496

[2] Torre LA Trabert B DeSantis CE Miller KD Samimi G Runowicz CD. Ovarian cancer statistics, 2018. CA Cancer J Clin. (2018) 68:284–96. 10.3322/caac.21456 29809280 PMC6621554

[3] Bast RC Jr Badgwell D Lu Z Marquez R Rosen D Liu J. New tumor markers: CA125 and beyond. Int J Gynecol Cancer. (2005) 15(Suppl. 3):2741–81. 10.1111/j.1525-1438.2005.00441.x 16343244

[4] Badgwell D Bast RC Jr. Early detection of ovarian cancer. Dis Markers. (2007) 23:397–410. 10.1155/2007/309382 18057523 PMC3851959

[5] Rosenthal AN Menon U Jacobs IJ. Screening for ovarian cancer. Clin Obstet Gynecol. (2006) 49:433–47. 10.1097/00003081-200609000-00004 16885651

[6] Buys SS Partridge E Black A Johnson CC Lamerato L Isaacs C. Effect of screening on ovarian cancer mortality: the Prostate, Lung, Colorectal and Ovarian (PLCO) cancer screening randomized controlled trial. JAMA. (2011) 305:2295–303. 10.1001/jama.2011.766 21642681

[7] Barnholtz-Sloan JS Schwartz AG Qureshi F Jacques S Malone J Munkarah AR. Ovarian cancer: changes in patterns at diagnosis and relative survival over the last three decades. Am J Obstet Gynecol. (2003) 189:1120–7. 10.1067/S0002-9378(03)00579-9 14586365

[8] NCCN Clinical Practice Guidelines in Oncology (NCCN Guidelines) -Ovarian Cancer Version 2. Fort Washington: National Comprehensive Cancer Network (2018). Available online at: https://www.nccn.org/professionals/physician_gls/pdf/ovarian.pdf (accessed July 19, 2018).

[9] Suh DH Chang SJ Song T Lee S Kang WD Lee SJ. Practice guidelines for management of ovarian cancer in Korea: a Korean Society of Gynecologic Oncology Consensus Statement. J Gynecol Oncol. (2018) 29:e56. 10.3802/jgo.2018.29.e56 29770626 PMC5981107

[10] Akhtar-Danesh N Elit L Lytwyn A. Temporal trends in the relative survival among patients diagnosed with ovarian cancer in Canada 1992–2005: a population-based study. Gynecol Oncol. (2011) 123:192–5. 10.1016/j.ygyno.2011.07.098 21855119

[11] Trétarre B Molinié F Woronoff AS Bossard N Bessaoud F Marrer E. Ovarian cancer in France: trends in incidence, mortality and survival, 1980–2012. Gynecol Oncol. (2015) 139:324–9. 10.1016/j.ygyno.2015.09.013 26383829

[12] Chan J Fuh K Shin J Cheung M Powell C Chen LM. The treatment and outcomes of early-stage epithelial ovarian cancer: have we made any progress? Br J Cancer. (2008) 98:1191–6. 10.1038/sj.bjc.6604299 18349835 PMC2359639

[13] Timmermans M Sonke GS Van de Vijver KK van der Aa MA Kruitwagen RFPM. No improvement in long-term survival for epithelial ovarian cancer patients: a population-based study between 1989 and 2014 in the Netherlands. Eur J Cancer. (2018) 88:31–7. 10.1016/j.ejca.2017.10.030 29179135

[14] Warren JL Harlan LC Trimble EL Stevens J Grimes M Cronin KA. Trends in the receipt of guideline care and survival for women with ovarian cancer: a population-based study. Gynecol Oncol. (2017) 145:486–92. 10.1016/j.ygyno.2017.03.016 28372872 PMC5489119

[15] Tan W Stehman FB Carter RL. Mortality rates due to gynecologic cancers in New York state by demographic factors and proximity to a Gynecologic Oncology Group member treatment center: 1979–2001. Gynecol Oncol. (2009) 114:346–52. 10.1016/j.ygyno.2009.03.033 19411096

[16] Chen C Markossian TW Silva A Tarasenko YN. Epithelial ovarian cancer mortality among Hispanic women: sub-ethnic disparities and survival trend across time: an analysis of SEER 1992–2013. Cancer Epidemiol. (2018) 52:134–41. 10.1016/j.canep.2017.12.003 29306788

[17] Surveillance Epidemiology and, End Results (SEER) Program (www.seer.cancer.gov) SEER^*^Stat Database: Incidence - SEER 18 Regs Custom Data (with additional treatment fields), Nov 2017 Sub (1973–2015 varying) - Linked To County Attributes - Total U.S., 1969–2016 Counties, National Cancer Institute, DCCPS, Surveillance Research Program, released April 2018, based on the November 2017 submission.

[18] Young JLJ Roffers SD Ries LAG Fritz AG Hurlburt AA (editors). SEER Summary Staging Manual - 2000: Codes and Coding Instructions. Bethesda, MD: National Institute of Health (2001). Available online at: https://seer.cancer.gov/tools/ssm/breast_femgen.pdf (accessed August 17, 2017).

[19] Kurman RJ Carcangiu ML Herrington CS Young RH WHO Classification of Tumours of Female Reproductive Organs. 4th ed Lyon: IARC (2014).

[20] Chen VW Ruiz B Killeen JL Coté TR Wu XC Correa CN. Pathology and classification of ovarian tumors. Cancer. (2003) 97:2631–42. 10.1002/cncr.11345 12733128

[21] Menon U Griffin M Gentry-Maharaj A. Ovarian cancer screening–current status, future directions. Gynecol Oncol. (2014) 132:490–5. 10.1016/j.ygyno.2013.11.030 24316306 PMC3991859

[22] McGuire WP Hoskins WJ Brady MF Kucera PR Partridge EE Look KY. Cyclophosphamide and cisplatin compared with paclitaxel and cisplatin in patients with stage III and stage IV ovarian cancer. N Engl J Med. (1996) 334:1–6. 10.1056/NEJM199601043340101 7494563

[23] Chi DS Liao JB Leon LF Venkatraman ES Hensley ML Bhaskaran D. Identification of prognostic factors in advanced epithelial ovarian carcinoma. Gynecol Oncol. (2001) 82:532–7. 10.1006/gyno.2001.6328 11520151

[24] van Altena AM Karim-Kos HE de Vries E Kruitwagen RF Massuger LF Kiemeney LA. Trends in therapy and survival of advanced stage epithelial ovarian cancer patients in the Netherlands. Gynecol Oncol. (2012) 125:649–54. 10.1016/j.ygyno.2012.02.033 22370602

[25] Bristow RE Tomacruz RS Armstrong DK Trimble EL Montz FJ. Survival effect of maximal cytoreductive surgery for advanced ovarian carcinoma during the platinum era: a meta-analysis. J Clin Oncol. (2002) 20:1248–59. 10.1200/JCO.2002.20.5.1248 11870167

[26] Averette HE Janicek MF Menck HR. The National Cancer Data Base report on ovarian cancer. American College of Surgeons Commission on Cancer and the American Cancer Society. Cancer. (1995) 76:1096–103. 10.1002/1097-0142(19950915)76:6<1096::AID-CNCR2820760626>3.0.CO;2-4 8625213

[27] Broekman K Jalving M van Tinteren H Sessa C Reyners A. Clinical benefit of controversial first line systemic therapies for advanced stage ovarian cancer – ESMO-MCBS scores. Cancer Treat Rev. (2018) 69:233–42. 10.1016/j.ctrv.2018.06.008 30098485

[28] Mirza MR Monk BJ Herrstedt J Oza AM Mahner S Redondo A. Niraparib maintenance therapy in platinum-sensitive, recurrent ovarian cancer. N Engl J Med. (2016) 375:2154–64. 10.1056/NEJMoa1611310 27717299

[29] Bellone S Buza N Choi J Zammataro L Gay L Elvin J. Exceptional response to pembrolizumab in a metastatic, chemotherapy/radiation-resistant ovarian cancer patient harboring a PD-L1-genetic rearrangement. Clin Cancer Res. (2018) 24:3282–91. 10.1158/1078-0432.CCR-17-1805 29351920 PMC6050068

[30] Vernooij F Heintz P Witteveen E van der Graaf Y. The outcomes of ovarian cancer treatment are better when provided by gynecologic oncologists and in specialized hospitals: a systematic review. Gynecol Oncol. (2007) 105:801–12. 10.1016/j.ygyno.2007.02.030 17433422

[31] Noone AM Lund JL Mariotto A Cronin K McNeel T Deapen D. Comparison of SEER treatment data with medicare claims. Med Care. (2016) 54:e55–64. 10.1097/MLR.0000000000000073 24638121 PMC4981219

